# Supplementary material for: Chromosome-Level Genome Assembly for the Angiosperm Silene conica
Source: Genome Biol Evol. 2023 Oct 20;15(11):evad192. doi: 10.1093/gbe/evad192 (PMC10630074; doi:10.1093/gbe/evad192)
Supplement: evad192_Supplementary_Data [file evad192_supplementary_data.pdf]

Table S1. Interspersed repeat content in the *S. conica* genome

	No. of Elements	Total Length (Mb)	Genome %
**LTR Retrotransposons**	**543123**	**472.03**	**54.77**
Ty1/Copia	157792	185.57	21.53
Gypsy/DIRS1	167710	181.10	21.01
Other	217621	105.36	12.23
**DNA Transposons**	**584735**	**152.27**	**17.67**
**Unclassified**	**49601**	**8.28**	**0.96**
**Total Interspersed Repeats**	**1177459**	**632.58**	**73.40**

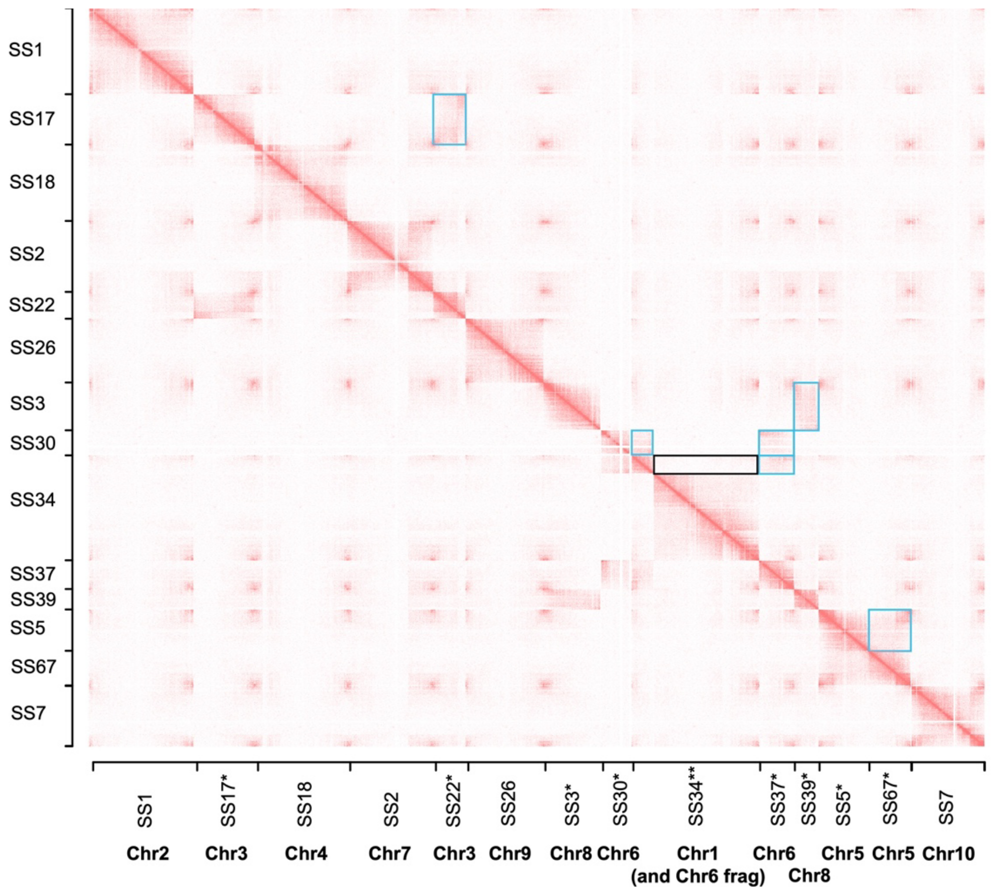

Figure S1. Heatmap generated with HiC-Pro and the HitC R package visualizing Hi-C interactions across the Bionano super-scaffolds (SS) from the *S. conica* genome. The chromosomes to which the scaffolds were eventually assigned are indicated on the x-axis. The outlined boxes above the primary diagonal highlight signal that led to joining scaffolds (teal boxes) or breaking apart a misassembly due to lack of Hi-C contacts within the scaffold (black box). The corresponding regions below the primary diagonal are left unhighlighted for visual comparison. Single asterisks (*) indicate Bionano scaffolds that were joined to form larger chromosome-level scaffolds. The double asterisk (**) indicates the misassembled Bionano SS34 scaffold that was subsequently separated into Chr1 and a portion of Chr6. Only 14 of the 16 Bionano scaffolds are visualized in this figure because the other two were too small and consisted of tandemly repeated ribosomal DNA regions. Those two scaffolds were joined with the other Chr6 scaffolds based on strength of Hi-C contact signal.

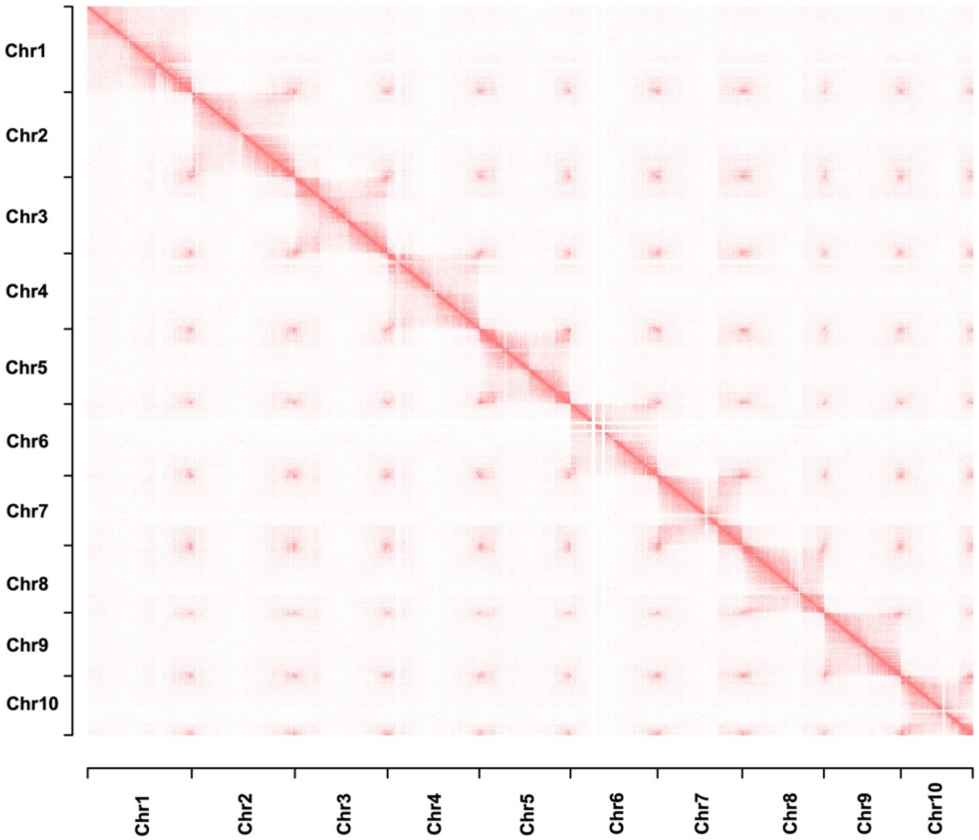

Figure S2. Heatmap generated with HiC-Pro and the HitC R package visualizing Hi-C interactions across the 10 chromosome-level scaffolds from the *S. conica* genome.

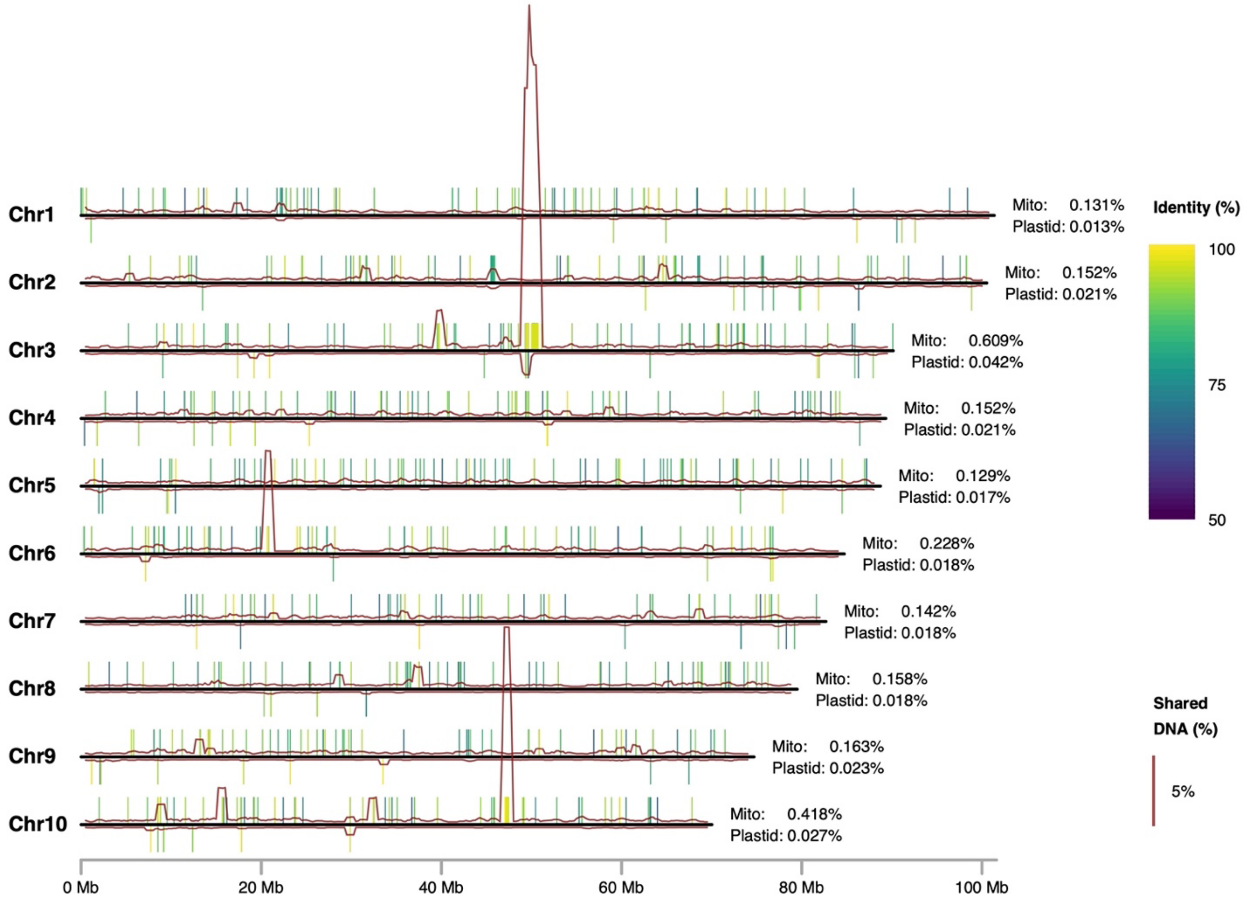

Figure S3. Summary of sequence content shared between the *S. conica* nuclear and cytoplasmic genomes. Tick marks above and below each nuclear chromosome indicate sequence content shared with the mitochondrial and plastid genomes, respectively, as identified by BLAST analysis (minimum hit length of 300 bp and e-value threshold of 1e-6). The color of each tick mark indicates the percent nucleotide identity of the BLAST hit. The red traces are from a sliding window analysis (1-Mb window size and 250-kb step size), indicating the percentage of sequence in the corresponding window that is shared with the mitochondrial genome (above the chromosome) or plastid genome (below the chromosome). Values on the right of each chromosome indicate the overall percentage of sequence shared with each cytoplasmic genome.
